# Transcriptional profiling of *Drosophila* S2 cells in early response to *Drosophila* C virus

**DOI:** 10.1186/1743-422X-10-210

**Published:** 2013-06-27

**Authors:** Fei Zhu, Haojie Ding, Binnian Zhu

**Affiliations:** 1 College of Animal Science and Technology, Zhejiang Agriculture and Forestry University, Hangzhou 311300, China

**Keywords:** Transcriptional profiling, *Drosophila* S2 cells, Early response, *Drosophila* C virus

## Abstract

**Background:**

The innate immune response like phagocytosis, encapsulation and antimicrobial peptide (AMP) production often occur in the early stage of host-pathogen interactions in *Drosophila melanogaster*. To investigate the *Drosophila* early immune response to *Drosophila* C virus, we characterized the DCV infection-response transcriptome of *Drosophila* Schneider 2 (S2) cells at one hour post inoculation.

**Method:**

The total RNA was extracted from treated S2 cells by using Trizol reagent and then analyzed by CapitalBio Corp for *Drosophila* GeneChip (Affymetrix) assay. Then the results of signaling pathway and protein interaction about these genes were analyzed by MAS 3.0 software.

**Results:**

Most significantly affected genes (656 genes) by DCV infection were regulated as the same way in inactivated DCV treatment, but inactivated white spot syndrome virus (WSSV) showed a different transcriptome. DCV infection up-regulated the expression levels of 275 genes and down-regulated that of 442 genes significantly and some affected genes were related to phagocytosis. DCV infection activated the JAK/STAT pathway by 1 hour post incubation. The Imd pathway was activated and transcriptional induction of antimicrobial peptides (AMPs) from this pathway was enhanced by 1 hour post incubation. But the Toll pathway was not activated like Imd pathway and the expression levels of AMPs from this pathway was reduced. In addition, most pattern-recognition receptors were inhibited and the antiviral RNAi pathway was not activated in the early stage of DCV infection.

**Conclusions:**

In conclusion, the present study demonstrates that DCV infection may activate phagocytosis, JAK/STAT pathway and Imd pathway in the early host-virus interactions. These results indicate that DCV is capable of activating or inhibiting some immune responses in the host cells and these changes would be vital for virus entry and replication.

## Background

The innate immune response of *Drosophila* is governed by numerous signaling pathways that trigger antimicrobial peptide (AMP) production, phagocytosis, melanization, and encapsulation to limit infection after exposure to microbes [1,2]. *Drosophila* C virus (DCV) is a non-occluded isometric virus which containing a positive sense RNA genome [3,4]. DCV is a natural pathogen of the model organism *D*. *melanogaster*, making it an ideal model system for studying invertebrate host-virus interactions [5]. The mechanisms of antiviral defense in *Drosophila* highlight the potential of the *D*. *melanogaster* model for studying antiviral innate immunity [6]. It is found that the Imd pathway is involved in the antiviral immune responses of *Drosophila*[7,8]. The Toll pathway is required for efficient inhibition of *Drosophila* X virus replication in *Drosophila* and constitutive activation of the pathway resulted in decreased viral titer [9]. Recently RNA interference (RNAi) was found to mediate innate antiviral immunity in *Drosophila*[10-12]. The JAK/STAT signaling pathway is reported to involve in the antiviral response of *Drosophila*[11-13]. However, many viruses always develop the ability of suppressing or evading host immune response. No evidence for the activation of the Toll, IMD or JAK/STAT pathways was found in *D*. *melanogaster* infected with the sigma virus (*Rhabdoviridae*) [14]. And dengue virus (DENV) may suppress immune responses at early infection stages before activating them at later time points in *Aedes aegypti*[15]. So it is very necessary to study the early immune response to pathogenic virus in host cells.

White spot syndrome virus (WSSV) is a bacilliform, enveloped double stranded DNA virus that causes viral diseases in shrimp [16]. To investigate early immune responses against DCV, we exposed *Drosophila* S2 cells to DCV, inactivated DCV (inDCV), and inactivated WSSV (inWSSV). We selected inWSSV as a treatment but not WSSV because WSSV was not phagocytosed by S2 cells like DCV and inDCV and induced very complicated early response. We investigated the transcriptional profile of virus-challenged *Drosophila* S2 cells using oligonucleotide DNA microarrays to identify the *Drosophila* early immune response to DCV. This results of this study contribute to the understanding of early immunologic defense responses in invertebrate hosts to viral challenge, and this study paves the way for further experiments which investigate the roles of genes and pathways in antiviral immunity.

## Results and discussion

### Genome-wide analysis of the *Drosophila* early immune response to DCV

**Figure 1:**
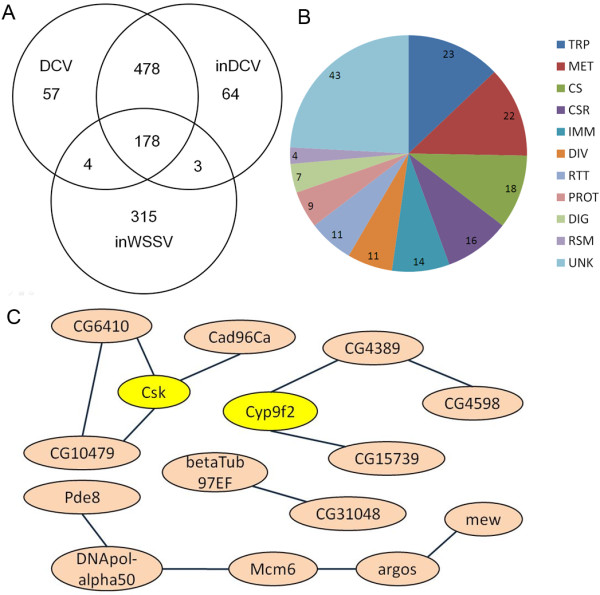
**Genome**-**wide analysis of the *Drosophila *early immune response to DCV. (A)** Venn diagrams of genes that changed expression in DCV, inDCV and inWSSV treatments. **(B)** Functional classification of 178 significantly regulated genes in DCV, inDCV and inWSSV treatments. Functional group abbreviations are as follows: UNK, unknown functions; TRP, transport; MET, metabolism; CS, cytoskeletal and structural; CSR, chemosensory reception; DIV, diverse functions; RTT, replication, transcription, and translation; PROT, proteolysis; DIG, blood and sugar food digestive; RSM, redox, stress and mitochondrion; IMM, immunity. **(C)** The pathway gene correlation of the 57 genes according to KEGG databases.

We investigate *Drosophila* S2 cells in early immune response to DCV to systematically dissect host functions important in responding to virus. Genome-wide analysis can be conducted easily in genetically tractable model hosts, such as *D*. *melanogaster*, and such analyses offer a new approach to identifying host genes required for host antiviral immunity. We conducted a genome-wide analysis of the *Drosophila* early immune response to DCV by using oligonucleotide microarrays. Our results show that DCV infection significantly up-regulated the expression levels of 275 genes and significantly down-regulated that of 442 genes at least 2-fold (Table 1). Some of these genes function as immunity signal transduction, antimicrobial peptides, pattern-recognition receptors and apoptosis (Table 2). Most of these affected genes (656 genes) were regulated commonly in DCV and inDCV treatment (Figure 1A). In the 656 affected genes, the most highly up-regulated 5 genes were Pherokine 3 (>50 fold, function as protein serine/threonine kinase activity), shaven baby (>28 fold, function as sequence-specific DNA binding), Matrix metalloproteinase 1 (>23 fold, function as metalloendopeptidase activity), Ribosomal protein S5b (>13 fold, function as RNA binding) and CG34330 (>13 fold, function as neurogenesis). And the most strongly down-regulated 5 genes were Hemese (>50 fold, function as negative regulation of lamellocyte differentiation), CG34003 (>30 fold, function as bacterial cell surface binding), nimrod C1 (>29 fold, function as defense response to bacterium), Projectin (>24 fold, function as structural constituent of cytoskeleton) and CG9616 (>21 fold). In addition, 178 genes were regulated commonly in DCV, inDCV and inWSSV treatments. The functional analysis shows that the 178 genes participated in diverse biological processes including transport, cellular metabolism, cytoskeleton regulation, chemosensory reception, diverse functions and so on (Figure 1B). In addition, 57 genes were significantly affected in *Drosophila* S2 cells at 1 hour post inoculation in cells infected with DCV but not in the other two treatments (Figure 1A). A total of 24 genes were up-regulated and 33 genes were down-regulated significantly (*P*<0.05). Gene ontology and KEGG analyses revealed that most of these genes were related to cellular metabolism. The similar result was found in *Aedes aegypti* cells with dengue virus infection [17]. Among the 57 genes, βTub97EF, DNApol-α50, Cyp9f2, and Csk were not directly related to cellular metabolism (Figure 1C). βTub97EF has GTPase activity and contributes to microtubule-based movement and phagosome conserved biosystem, and it is linked with CG31048 which participate in activation of Rac GTPase activity. DNApol-α50 has DNA polymerase activity and contributes to DNA replication and synthesis of RNA primer, and it is linked with Pde8 (3',5'-cyclic-AMP phosphodiesterase activity), Mcm6 (contributes to 3'-5' DNA helicase activity), argos (receptor antagonist activity) and mew (cell adhesion molecule binding). Cyp9f2 is an age-regulated gene which anticipates in oxidation-reduction process, and it is linked with CG4389 (enoyl-CoA hydratase activity), CG4598 (dodecenoyl-CoA delta-isomerase activity) and CG15739 (4-nitrophenylphosphatase activity) [18]. βTub97EF, Cyp9f2 and DNApol-α50 were down-regulated very significantly (*P*<0.01) in DCV treatment but not in the other two treatments. C-terminal Src kinase (Csk) is the major inhibitor of Src signaling, and it is linked with CG6410 (phosphatidylinositol binding), CG10479 (unknown function) and Cad96Ca (protein tyrosine kinase activity). In this study, Csk was up-regulated very significantly (*P*<0.01) in DCV treatment but not in the other two treatments. Csk functions with Src-family kinases to negative regulate cell proliferation and positive regulate apoptosis [19-24]. Endocytosis or phagocytosis is the key step in the interaction between virus and host cells, and virus could utilize them to entry and infect host cells [25,26]. So these genes induced by DCV infection may involved in phagocytosis of host cells in 1 hour post inoculation.

**Table 1:** The list of DCV infection affected genes

**The number of affected genes**	**Gene symbol**
275 up-regulated genes	18w, a, Ald, alpha-Adaptin, alpha-Est1, alphaPS4, alphaPS5, AnnIX, alphaTub84D, aPKC, Asator, Ast, Atpalpha, AttA, AttC, AttD, b, B52, Best1, bnl, brp, bves, CaMKII, Ccn, Cct1, Cdk5, CecB, CecC, CG10011, CG10103, CG10337, CG10581, CG10630, CG10641, CG10657, CG10702, CG10962, CG1124, CG11353, CG11671, CG11779, CG11790, CG11791, CG11825, CG11897, CG12014, CG12054, CG12112, CG12290, CG12418, CG12477, CG12883, CG12896, CG13078, CG13196, CG13248, CG13335, CG1340, CG13482, CG14015, CG14085, CG14322, CG14340, CG14545, CG14567, CG14801, CG14879, CG15097, CG15308, CG15543, CG15673, CG1600, CG16717, CG16718, CG16833, CG17599, CG17660, CG17681, CG18528, CG18557, CG18643, CG18769, CG30108, CG30115, CG30281, CG30421, CG30466, CG30502, CG31012, CG31323, CG31324, CG31431, CG31522, CG31523, CG3168, CG31778, CG32048, CG32066, CG32170, CG32206, CG32207, CG32313, CG32512, CG32982, CG33099, CG3348, CG34330, CG34349, CG34360, CG34383, CG34404, CG3788, CG3884, CG42327, CG42348, CG4455, CG4570, CG4629, CG4726, CG5174, CG5246, CG5346, CG5535, CG5758, CG5919, CG6051, CG6125, CG6231, CG6330, CG6357, CG6498, CG6767, CG7056, CG7251, CG7510, CG7720, CG7778, CG7794, CG7816, CG7841, CG7888, CG8008, CG8046, CG8177, CG9119, CG9222, CG9238, CG9312, CG9626, CG9641, CG9663, CG9812, CG9932, cher, chn, chrb, Cortactin, Cpr67Fa1, Csk, Cyp4g1, DAAM, dally, Dhap-at, drl, Ets21C, ewg, fra, Gadd45, GlcAT-S, Gli, Gp150, Gr94a, gsb, h, Hip1, Hsp22, Hsp70Aa, Hsp70Ba, Hsp70Bc, ifc, ImpL2, ImpL3, insc, inx2, Irk3, jar, JhI-21, Jupiter, kay, kel, KP78b, KrT95D, lcs, Lerp, Lis-1, Lmpt, loco, LpR2, Luna, Mctp, Mf, Mmp1, moody, Mpk2, mthl2, Mtk, MtnA, Myo28B1, Myo31DF, mys, nahoda, nau, nes, Nhe3, nkd, Nrt, Oatp30B, Obp44a, Or19a, ovo, Pabp2, pain, Pak, path, Pde8, Phk-3, pirk, Pka-C3, PKD, pot, ppk10, Prx2540-2, puc, Pvf2, Rel, Rep, Rgn, RhoGEF3, RhoL, RN-tre, rogdi, RpS5b, rtGEF, scarface, sdk, shn, SIP3, slgA, Socs36E, Sox14, spir, Stam, stv, Su(dx), tamo, Thor, tmod, Tom34, Trc8, Tsp, Tsp42Eg, tty, Ugt36Bb, upd2, upd3, vfl, viaf, vir-1, Vrp1, WASp, wun, wun2, yellow-b, zfh1, zpg
442 down-regulated genes	Ac13E, Ac76E, Acer, Acox57D-d, Act79B, Adk3, Amph, Ance-5, arg, armi, Atet, att-ORFA, aub, Bc, Best4, betaTub97EF, bgm, bmm, bt, by, Cad96Ca, CG10026, CG10073, CG10126, CG10131, CG10184, CG10205, CG10249, CG10336, CG10469, CG10479, CG10512, CG10550, CG10660, CG10764, CG10863, CG11063, CG11134, CG11147, CG11151, CG11319, CG11347, CG11395, CG11400, CG11638, CG11668, CG11686, CG11739, CG11943, CG12140, CG12262, CG12340, CG12512, CG12702, CG12744, CG12825, CG12970, CG13085, CG13116, CG13377, CG13559, CG13631, CG13641, CG13654, CG13707, CG13794, CG13822, CG13877, CG13897, CG14033, CG14141, CG14215, CG14216, CG14225, CG14511, CG14615, CG14619, CG14629, CG14741, CG14787, CG14803, CG14806, CG14856, CG14872, CG14933, CG14990, CG1503, CG15043, CG15161, CG15202, CG15333, CG15658, CG15739, CG15818, CG15820, CG15917, CG1607, CG1623, CG1628, CG1637, CG1648, CG16700, CG16712, CG16713, CG1674, CG16947, CG1702, CG17029, CG17032, CG17167, CG17270, CG17322, CG17323, CG17350, CG17549, CG17597, CG17839, CG17928, CG18446, CG18522, CG18549, CG18563, CG18622, CG2003, CG2052, CG2444, Cg25C, CG2893, CG30017, CG30069, CG30085, CG30090, CG30104, CG30148, CG30217, CG30269, CG30273, CG30345, CG30359, CG3036, CG30377, CG30460, CG30463, CG30479, CG30492, CG31048, CG31075, CG31145, CG31274, CG31313, CG31326, CG31454, CG31477, CG31601, CG31607, CG31674, CG31675, CG3184, CG31886, CG3191, CG31974, CG31999, CG32017, CG32085, CG32091, CG3224, CG32306, CG32320, CG32354, CG32364, CG3246, CG32582, CG3259, CG32613, CG32647, CG32700, CG32812, CG33225, CG33252, CG33275, CG33465, CG3402, CG34331, CG34398, CG34436, CG3505, CG3635, CG3829, CG3831, CG3857, CG3902, CG40160, CG4019, CG40244, CG41265, CG42259, CG42296, CG42345, CG42358, CG42369, CG42394, CG4250, CG42611, CG4325, CG4351, CG4389, CG4398, CG4484, CG4576, CG4598, CG4615, CG4666, CG4733, CG4928, CG4949, CG5080, CG5167, CG5191, CG5322, CG5381, CG5397, CG5455, CG5707, CG5731, CG5853, CG5895, CG5955, CG5958, CG5973, CG6045, CG6188, CG6199, CG6208, CG6232, CG6289, CG6410, CG6426, CG6639, CG6687, CG6812, CG6836, CG6951, CG7059, CG7083, CG7091, CG7120, CG7149, CG7255, CG7280, CG7320, CG7322, CG7358, CG7458, CG7607, CG7777, CG7781, CG7966, CG7985, CG7995, CG7997, CG8066, CG8080, CG8097, CG8112, CG8157, CG8211, CG8213, CG8353, CG8398, CG8399, CG8451, CG8501, CG8586, CG8668, CG8788, CG9008, CG9098, CG9117, CG9232, CG9331, CG9338 , CG9416, CG9463, CG9505, CG9541, CG9577, CG9616, CG9624, CG9691, CG9973, CG9989, cpo, Cpr49Ac, Cpr65Au, Cpr97Eb, CPTI, CREG, Cyp12c1, Cyp12d1-d, Cyp18a1, Cyp28a5, Cyp28d1, Cyp4ac1, Cyp4d2, Cyp4s3, Cyp6a13, Cyp6a14, Cyp6a21, Cyp9f2, Cyp9h1, Cys, Dh, dj-1beta, DNApol-alpha50, DNaseII, dpp, dpr17, drpr, eater, edl, egr, Ela, fan, fbp, fng, fru, fz2, GLaz, glob1, Glt, grh, GstD4, GstD5, GstD6, GstD7, He, Hil, hoe1, Hr51, Hsp60B, htl, if, ine, Invadolysin, inx3, Irp-1B, Jheh3, Kap-alpha3, l(3)neo38, lectin-24A, lectin-28C, lin-28, Lip4, Lkr, lox, mAcR-60C, mav, mbc, Mcm6, mew, mex1, mspo, Myd88, MYPT-75D, nAcRalpha-30D, Nep4, NetB, Nha2, nimB2, nimB3, nimB4, nimB5, nimC1, nimC2, Oat, Oatp33Ea, Obp18a, Obp99a, Obp99c, obst-A, olf186-M, out, Pde6, Pdk, PGRP-LE, Pka-R2, PNUTS, prc, Prestin, Pxn, pyd, pyd3, qtc, r-cup, rdgB, rg, Rgk1, Rph, Rpt3R, ry, scpr-A, scu, shf, Sip1, Sk1, skpB, sls, sn, snk, Sp212, sqz, Sr-CI, Sry-alpha, stnA, Strn-Mlck, su(r), Sucb, Sur, sut1, Taf12L, TepI, topi, TotA, troll, Tsp29Fa, Tsp2A, Tsp5D, TwdlE, twi, Ubc84D, Ugt35a, Ugt36Bc, Ugt86Dd, Vago, veil, vkg, W, wnd, y, yellow-f2, yellow-h, yip2

**Table 2:** **Expression profiles of selected important immune genes revolved in early immune response to DCV**, **inDCV and inWSSV**

**Gene (GenBank accession number)**	**DCV**	**inDCV**	**inWSSV**
pattern-recognition receptors			
PGRP-LE (AAF48519)	0.41	0.41	1.23
PGRP-LB (AAF54643)	1.51	1.16	2.71
PGRP-LF (AAF50301)	1.82	1.65	2.14
PGRP-SB1 (AAF49420)	1.81	1.64	2.09
Sr-CI (AAF51042)	0.15	0.13	1.22
Sr-CIII (AAN11166)	0.49	0.51	1.36
Eater (AAF56664)	0.32	0.32	0.65
TepI (AAF53490)	0.22	0.21	1.07
Lkr (AAF50775)	0.19	0.22	0.25
NimB2 (AAN10861)	0.12	0.10	0.29
NimB3 (ABC65899)	0.03	0.03	0.12
NimB4 (AAF53361)	0.12	0.10	0.33
NimB5 (AAF53363)	0.07	0.09	0.32
NimC1 (AAF53364)	0.03	0.03	0.10
NimC2 (AAF53366)	0.41	0.34	0.33
Antimicrobial peptides/protein			
Attacin-A (AAF58215)	2.78	2.32	2.51
Attacin-C (AAM68570)	5.37	4.87	7.00
Attacin-D (AAF55446)	5.41	5.08	5.48
Andropin (AAF57024)	1.59	1.73	0.75
Cecropin (AAF57025)	7.94	8.14	1.28
Cecropin-B (AAF57027)	9.55	9.48	12.24
Drosomycin (AAF47767)	0.52	0.50	2.14
Drosocin (AAF58216)	1.29	1.24	2.19
Defensin (AAF58855)	0.53	0.43	0.84
Metchnikowin (AAF58139)	2.97	2.79	2.68
lectin24A (AAF51070)	0.39	0.49	0.87
lectin28C (AAF52570)	0.44	0.45	0.76
Immunity signal transduction			
bnl (AAF55701)	2.50	2.92	0.45
Daam (AAF45600)	2.15	2.07	2.23
Dally (AAF50358)	2.41	2.24	2.22
bt (AAF55701)	0.04	0.04	0.07
mspo (AAF58219)	0.06	0.06	1.07
yellow-h (AAF59358)	0.07	0.08	0.16
prc (AAF49980)	0.05	0.06	0.36
Bc (AAF53744)	0.06	0.06	0.85
TotA (AAN13840)	0.07	0.06	0.38
Fz2 (AAF49184)	0.43	1.04	0.41
Myd88 (AAF58953)	0.39	0.39	1.16
Hsp60B (AAF51467)	0.47	0.41	1.94
Hsp70Aa (AAN13535)	2.72	4.55	0.64
Hsp70Bc (AAG22149)	2.43	5.04	0.88
Rel (AAF54333)	4.65	4.61	4.03
Mpk2 (AAF56244)	2.36	2.32	2.24
Rgk1 (AAF57577)	0.26	0.33	0.37
Mav (AAF59328)	0.33	0.30	0.48
upd3 (AAX52505)	2.70	3.07	4.33
upd2 (AAF48815)	5.23	6.42	1.74
Vago (AAF47993)	0.26	0.21	0.37
Vir-1 (AAF53185)	8.70	8.92	5.81
Vrp1 (AAF46800)	6.72	7.31	2.49
Ets21C (AAF51484)	3.38	4.03	3.58
dpr17 (AAF54750)	0.10	0.10	0.27
He (AAN10862)	0.02	0.02	0.18
Moody (AAF45709)	2.48	2.51	2.04
Pirk (AAF46746)	10.89	10.62	10.70
CG33275 (AAS65060)	0.07	0.08	0.39
CG9098 (AAF52322)	0.14	0.14	0.32
CG32354 (AAF50418)	0.27	0.30	2.55
CG4019 (AAF47036)	0.12	0.11	0.49
Apoptosis			
AnnIX (AAF55841)	2.54	2.45	2.04
Sk1 (AAF48045)	0.28	0.26	0.44
Stv (AAF49809)	3.79	3.74	2.54
Viaf (AAF49974)	2.66	2.67	2.28
W (AAF49270)	0.22	0.21	0.34
Out (AAF48949)	0.43	0.37	0.44
CG3829 (AAF47310)	0.31	0.29	0.46

The gene expression levels were compared with that of control (S2 cells without any treatment). The control’s was designated as 1.

### JAK/STAT pathway is involved in antiviral immunity in *Drosophila*

**Figure 2:**
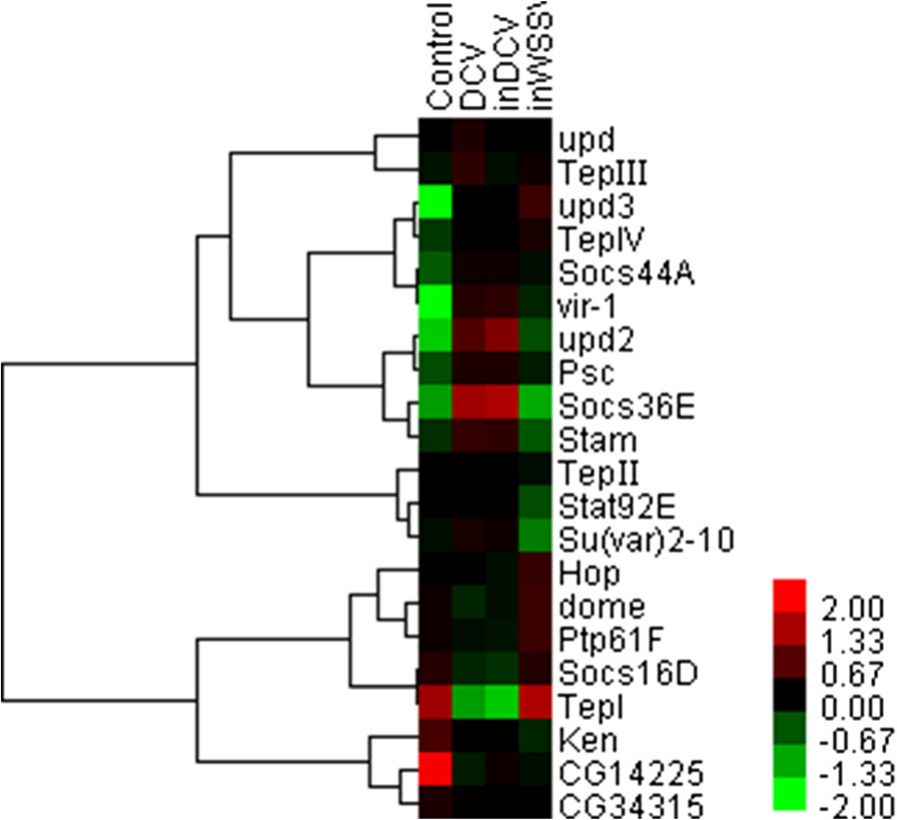
**Hierarchical cluster analysis of DNA microarray data obtained from 21 genes in JAK/STAT pathway.**

As a reporter of *Drosophila* responsible for DCV infection, virus-induced RNA 1 (vir-1) is one of genes resulting from JAK/STAT signaling pathway which is not induced by pathogenic bacteria or fungi [13,27]. Expression of Vir-1 was not modified by many stresses, such as heat shock, cold shock, mechanical pressure, dehydration or ultraviolet irradiation [27]. Therefore the Vir-1 gene specifically functions in the antiviral immune response of *Drosophila*[5]. The results of this study showed that Vir-1 was significantly up-regulated (*P*<0.01) in all three treatments, suggesting the involvement of JAK/STAT pathway in antiviral responses of *Drosophila* (Figure 2). Furthermore, Vir-1 was significantly up-regulated (*P*<0.01) in response to WSSV (a non-pathogenic virus of *Drosophila*) as it was in responses to DCV (Figure 2). Our results show that upd2, Socs36E and Stam were up-regulated very significantly (*P*<0.01), and TepI was down-regulated very significantly (*P*<0.01) in pathogenic virus treatments (DCV and inDCV) but not in non-pathogenic virus treatment (inWSSV) (Figure 2). According to our data in this study, upd2, Socs36E, Stam and TepI have special antiviral immunity to pathogenic virus like DCV. But upd3 and vir-1 were up-regulated significantly (*P*<0.01) in all three treatments (Figure 2). The cytokine unpaired 3 (upd3) has been previously understood to activate the JAK/STAT pathway in hemocytes [28,29]. The data indicate that upd3 and vir-1 belong to the common antiviral immunity in *Drosophila*. A previous study showed that *Drosophila* did not mount an immune response controlled by known immune pathways against the sigma virus [14]. However, we found that the JAK/STAT pathway was activated by DCV infection at 1 hour post incubation.

### Toll pathway is involved in early response to *Drosophila* C virus

**Figure 3:**
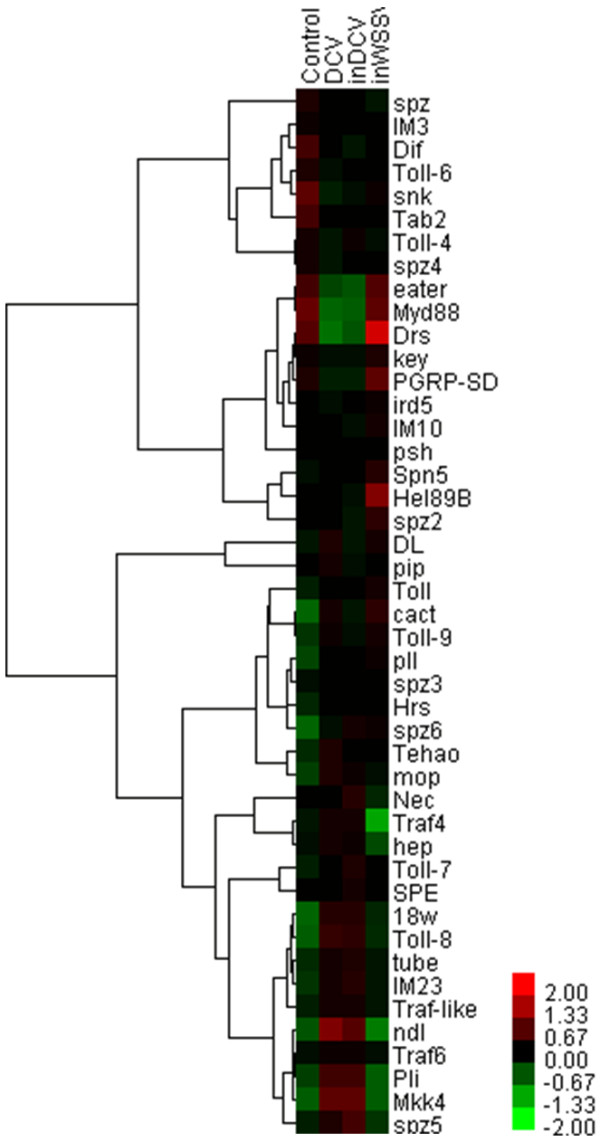
Hierarchical cluster analysis of DNA microarray data obtained from 45 genes in Toll pathway.

Previously, it was shown that the *Drosophila* X virus (DXV) activates the Toll pathway, and flies that are deficient for the Toll pathway transcription factor Dif are more susceptible to DXV infection [9]. Another study showed that Dif deficient flies exhibit the same sensitivity to DCV infection as wild-type flies [30]. In this study, DCV infection down-regulated the expression levels of Dif at one hour post infection. In Toll pathway, MyD88 and eater were down-regulated significantly (*P*<0.01), and Mkk4, ndl, 18w, Toll-8 and Pli were up-regulated significantly (*P*<0.01) in pathogenic virus treatments (DCV and inDCV) but not in non-pathogenic virus treatment (inWSSV) (Figure 3). The data indicate that these genes have special immunity to pathogenic virus like DCV. Interestingly, Drs (Drosomycin) was down-regulated significantly (*P*<0.01) in pathogenic virus treatments (DCV and inDCV) but was up-regulated significantly (*P*<0.01) in non-pathogenic virus treatment (inWSSV). Drosomycin (Drs) gene encodes a 44-residue inducible antifungal peptide, Drosomycin, in *Drosophila melanogaster*[31]. Because the Toll pathway is so important for antiviral immunity of *Drosophila*, DCV may inhibit the key factors of this pathway to evade humoral and cellular responses of host cells.

### Imd pathway is involved in early response to *Drosophila* C virus

**Figure 4:**
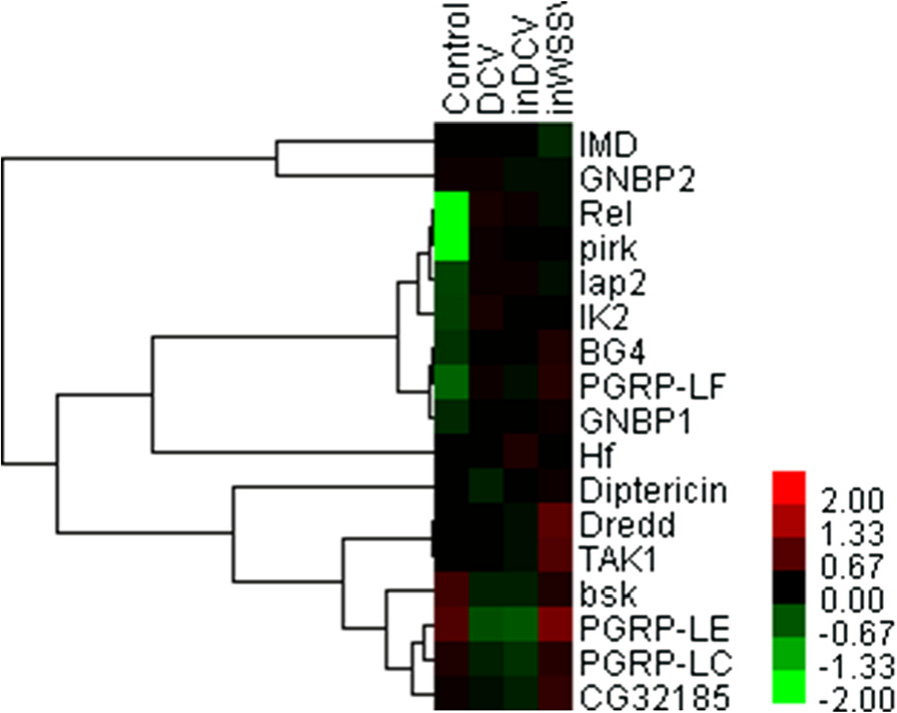
Hierarchical cluster analysis of DNA microarray data obtained from 17 genes in Imd pathway.

In the Imd pathway, Rel, pirk and PGRP-LF were up-regulated very significantly (*P*<0.01) in all three treatments (Figure 4). PGRP-LE and PGRP-LC, PGRP family member, are required for activation of the Imd pathway in response to Gram-negative bacterial infections [32]. In this study, PGRP-LE was down-regulated very significantly (*P*<0.01), and PGRP-LC was down-regulated in pathogenic virus treatments (DCV and inDCV) but not in non-pathogenic virus treatment (inWSSV) (Table 2). The NF-κB-like transcription factor Relish is the ultimate target of the Imd pathway, which regulates the expression of a battery of genes encoding antimicrobial peptides (AMPs)-like Attacins [33,34]. AMP genes have been shown to be induced in response to viral infection at levels similar to those observed during *E*. *coli* infection [9]. In this study, Attacin-A, Attacin-C, Attacin-D, Cecropin-B and Metchnikowin were very significantly up-regulated (*P*<0.01) in all three treatments (Table 2). The results suggested that the Imd pathway was activated in DCV treatment and that the Imd pathway may be involved in the antiviral immunity in *Drosophila*. However, enhanced expression of single AMPs did not alter resistance to viral infection or viral titer levels, suggesting that the main antiviral response is cellular rather than humoral [9]. DENV infection also down-regulated the expression levels of numerous immune signaling molecules and AMPs in *Aedes aegypti* cells [15,17]. In this study, Drosomycin, Defensin, lectin24A and lectin28C were down-regulated very significantly (*P*<0.01) in pathogenic virus treatments (DCV and inDCV) but not in non-pathogenic virus treatment (inWSSV) (Table 2). The data revealed that Drosomycin, Defensin, lectin24A and lectin28C may play more important role in antiviral immunity than other AMPs.

### RNAi pathway was not activated in early response to *Drosophila* C virus

Previous studies have shown that RNA interference played a critical role in the control of viral infections in *Drosophila* and Ago2, Ars2, Dcr-2 and R2D2 as the core antiviral RNAi machinery [10,35,36]. However, the relative expression of Ago1, Ago2, Ars2, Dcr-1, Dcr-2, R2D2 and Drosha, which are important to antiviral RNAi pathway in *Drosophila*, remained stable in all three experimental groups (data not shown). The antiviral RNAi pathway was not activated in S2 cells by 1 hour post incubation with DCV or inWSSV. Virus infection in *Drosophila* initiates a specific transcriptional response, including the induction of Vago, a recently identified antiviral molecule that is required to restrict viral replication in flies [37]. In this study, Vago was significantly down-regulated (*P*<0.01) in all three treatments (Table 2). The data indicate that the antiviral RNAi pathway was not induced in S2 cells at 1 hour post incubation with DCV or inWSSV. Previous studies also showed that DCV encodes a dsRNA-binding protein, DCV-1A, which suppresses RNA silencing in *Drosophila*[38,39].

**Figure 5:**
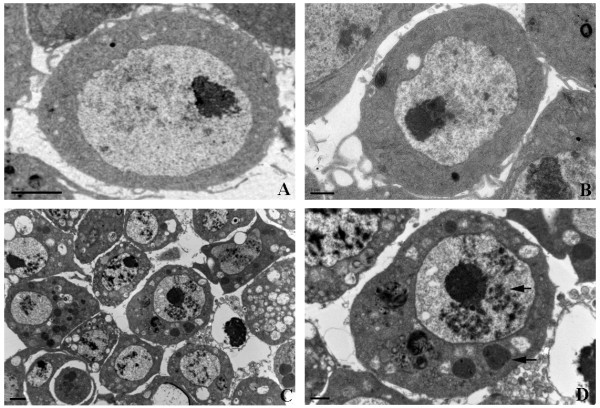
**Transmission electron microscopy (TEM) of DCV**-**infected S2 cells. (A)** Normal S2 cells, Bar=2 μm. **(B)** DCV-infected S2 cells at one hour post infection, Bar=1 μm. **(C)** DCV-infected S2 cells at one day post infection, Bar=2 μm. **(D)** Enlarge of one DCV-infected S2 cell at one day post infection, Bar=1 μm. The arrows indicate the typical morphological changes of S2 cell caused by DCV infection.

In *Drosophila*, invading pathogens can encounter humoral and cellular responses that utilize pattern-recognition receptors to identify pathogen-associated molecular patterns on the immune cell surface [1,2]. In this study, PGRP-LE, Sr-CI, Sr-CIII, Eater, TepI were down-regulated significantly (*P*<0.01) in pathogenic virus treatments (DCV and inDCV) but not in non-pathogenic virus treatment (inWSSV) (Table 2). And Lkr, NimB2, NimB3, NimB4, NimB5, NimC1, NimC2 were down-regulated significantly (*P*<0.01) in all three treatments (Table 2). The results indicate that DCV may escape the recognition of host immunity by inhibition of pattern-recognition receptors on S2 cell surface in the early stage of infection. Phagocytosis is the early initiated innate immunity in *Drosophila* cells, so it is very important for antiviral immunity [40,41]. DCV infection may activate or utilize phagocytosis immunity of host cells by 1 hour post inoculation because phagocytosis related genes (Csk and βTub97EF) were up-regulated significantly (*P*<0.01) by DCV infection only. TEM results also showed that no difference was found in DCV-infected S2 cells at one hour post infection, but DCV infection caused large harm to S2 cells at one day post infection (Figure 5). DCV infection activated the JAK/STAT pathway and the Imd pathway in the early host-virus interaction. But the Toll pathway was not activated by DCV infection in the early host-virus interaction and the expression levels of AMPs from this pathway was down-regulated. And the antiviral RNAi pathway was not activated by DCV infection in the early host-virus interaction. Our results indicate that DCV actively suppresses activation of some immune pathways in *Drosophila* cell lines. This work contributes to the understanding of the early immunologic defense responses in invertebrate hosts to viral challenge, and it paves the way for further experiments that investigate the roles of genes and pathways in antiviral immunity.

## Materials and methods

### Maintenance of *Drosophila* S2 cell line and treatment

*Drosophila* S2 cells were cultivated at 28°C in Schneider’s *Drosophila* medium (Ivitrogen, USA) supplemented with 10% fetal bovine serum (Gibco, USA). DCV was inoculated in S2 cells at a multiplicity of infection (MOI) of 1 for 4 days and collected for purification as described before [4]. Then S2 cells were infected with purified DCV at a multiplicity of infection (MOI) of 1. DCV at a multiplicity of infection (MOI) of 1 was UV-inactivated by exposure to a total of 12, 000 mJ UV light (5×3 min) as inactivated DCV (inDCV), and then S2 cells were inoculated with UV-inactivated DCV. The WSSV were purified from WSSV-infected shrimp according to the previous methods [42]. The WSSV virions were UV-inactivated by exposure to a total of 12, 000 mJ UV light (5×3 min). Subsequently the inactivated WSSV virions (1 × 10^7^ copies/mL) were inoculated in S2 cells (1 × 10^6^ cells/mL). After one hour, the S2 cells were collected and subjected to oligonucleotide microarray.

### Analyses of mRNA expressions with oligonucleotide microarray

The total RNA was extracted from treated S2 cells by using Trizol reagent (Invitrogen, USA) according to the manufacturer’s instructions. The total RNA samples were then analyzed by CapitalBio Corp for *Drosophila* GeneChip (Affymetrix) assay. And each treatment has 3 biological replicates that were measured by this way. Gene expression analysis was performed by using the Affymetrix (Santa Clara, CA, USA) *Drosophila* GeneChip, using the laboratory methods in the Affymetrix GeneChip expression manual. Gene expression analysis was performed using triple arrays and triple independent mRNA samples for each treatment. Microarray data were analyzed by using Bio MAS (molecule annotation system) 3.0 software (CapitalBio Corporation, Beijing, China). Using the criterion of cutoff limitation as a fold change ≥ 2 or ≤0.5 and q-value ≤ 5%, differential expression genes were screened and clustered.

### Biological pathway analysis

Through array analysis, the commonly altered genes were screened from DCV, and WSSV treatments. The selected genes were further analyzed in the context of Gene Ontology (GO) biological process and Kyoto Encyclopaedia of Genes and Genomes (KEGG) biological pathway. Then the results of signaling pathway and protein interaction about these genes were analyzed by MAS 3.0 software. To reveal the functions of predicted target genes, we used the ontology classification of genes based on gene annotation and summary information available through DAVID (Database for Annotation, Visualization and Integrated Discovery).

### Transmission electron microscopy assay

The S2 cells were pelleted and fixed in the fixative containing 2% paraformaldehyde and 2% glutaraldehyde in 0.1 M sodium cacodylate buffer (pH 7.4) for 18 h at room temperature. Each sample was washed three times with 0.1 M sodium cacodylate buffer at room temperature. Then the sample was postfixed with 2% osmium tetroxide in 0.1 M sodium cacodylate buffer with constant rotation for 1h, followed by washes three times using 0.1 M sodium cacodylate buffer at room temperature. The sample was stained with 2% uranyl acetate in 0.2 M sodium acetate buffer (pH 5.2) for 1 h at room temperature and subsequently washed three times with 0.2 M sodium acetate buffer at room temperature. The sample was dehydrated in an ascending acetone series (50, 60, 70, 80, 90, 95, and 100%) and then in 100% propylene oxide for 10 min with constant rotation at room temperature. After infiltration of sample with EMBED 812/Araldite 502 resin at room temperature, sections were prepared in a Reichert Ultracut OMU3 microtome (Leica, Germany) at 100 nm thickness, followed by staining with 2% uranyl acetate/70% methanol. The images were collected on a Hitachi 7650 transmission electron microscope (Hitachi, Japan) operating at 70 kV.

### Statistics

Statistical significance was determined by Student's t-test (two-tailed distribution with a two sample equal variance). *P*-values of less than 0.05 were considered significant and less than 0.01 were considered very significant.

## Competing interests

The authors declare that they have no competing interests.

## Authors’ contributions

ZF designed the experiments. ZF, DHJ and ZBN carried out the experiments. ZF, DHJ and ZBN analyzed the data, ZF wrote the paper. All authors read and approved the final manuscript.
